# Trends in Diabetes Mellitus and Associated Mental Disorders‐Related Mortality (1999–2023): A CDC WONDER Database Analysis

**DOI:** 10.1002/brb3.70723

**Published:** 2025-08-04

**Authors:** Syed Tawassul Hassan, Muhammad Shaheer Bin Faheem, Yasmeen Sufi, Anushah Nadeem, Wajeeha Siddiqui, Dur E. Sameen, Amna Kaleem Ahmed, Faiza Nasir, Sumaya Samadi

**Affiliations:** ^1^ Karachi Medical and Dental College Karachi Pakistan; ^2^ Karachi Institute of Medical Sciences, KIMS Karachi Pakistan; ^3^ Jinnah Medical & Dental College Karachi Pakistan; ^4^ Kabul University of Medical Sciences “Abu Ali Ibn Sina” Kabul Afghanistan

**Keywords:** age‐adjusted mortality rates, associated mental and behavioral disorders, CDC WONDER, diabetes mellitus

## Abstract

**Objectives:**

Diabetes mellitus (DM) has a strong bidirectional relationship with mental disorders, collectively increasing the risk of mortality. We aim to analyze the mortality trends related to DM and associated mental disorders while exploring demographic and regional disparities in the United States from 1999 to 2023.

**Methods:**

Death records were obtained from the Centers for Disease Control and Prevention‐Wide‐ranging ONline Data for Epidemiologic Research (CDC WONDER) database from 1999 to 2023. Crude and age‐adjusted mortality rates (AAMRs), measured per 100,000 people, were calculated and standardized to the 2000 US standard population. Annual percent changes (APCs) in AAMRs and crude mortality rates (CMRs) were computed through the Joinpoint regression program and data stratification was done based on sex, race, age, and geographical regions.

**Results:**

A total of 1,332,198 death records indicated the presence of both DM and mental disorders. AAMR increased fourfold from 1999 (9.5) to 2023 (40.4) per 100,000 population. Notable increase shifts were noted in AAMR across different periods between 1999 and 2021 (APC: 1999–2005: 14.98; 2005–2018: 2.92; 2018–2021: 8.98), after which it declined sharply until 2023 (APC: −6.42). Males (36.8) had elevated AAMRs in comparison to females (22.4). Non‐Hispanic (NH) American Indians contributed to most of the AAMR (39.8). CMR was 11‐fold higher in older adults as compared to those aged between 35 and 64 years. Regionally, AAMRs in nonmetropolitan areas (35.9) and Midwest regions (34.7) were the highest.

**Conclusions:**

Further research and targeted interventions are needed for in‐depth evaluation of these rising mortality trends to find the root cause and lower the burden of DM and associated mental disorders.

## Graphical abstract

This study highlights rising diabetes‐ and mental disorders‐related mortality with sharp increases in mortality rates during the COVID‐19 pandemic. Disparities across age, sex, race, and region persist, stressing the need for localized interventions with proper resource allocation among high‐risk populations.

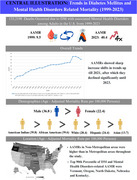

## 1 Introduction

Diabetes mellitus (DM) is defined as a metabolic disorder either due to insulin deficiency or insulin resistance causing elevated levels of glucose (hyperglycemia) (Davey et al. 2018). It was responsible for approximately 1.5 million deaths in 2019, making it one of the top 10 leading causes of death and a significant public health challenge across the globe, contributing to the overall disease burden (Xu et al. 2024). People with DM not only experience different metabolic disorders but also face a higher risk for cancer and noncommunicable diseases, including mental disorders, as compared to the general population. DM‐associated mortality is responsible for approximately 35.6% of deaths from noncommunicable diseases and 2.7% of all deaths worldwide (Xu et al. 2024). DM is one of the most common causes of morbidity and death in the United States, causing approximately 440,000 deaths among US adults in 2019 (Thomson et al. 2024). Studies suggest that DM‐related complications significantly increase the risk of developing mental disorders, with a hazard ratio (HR) ranging from 1.9 to 2.9 (*p* < 0.05), and the risk was higher in older age groups (Watanabe et al. 2024). Conversely, mental disorders can worsen DM‐associated complications among DM patients, with an HR between 1.4 and 2.5 (*p* < 0.05), with the highest risk observed in individuals aged 0–19 years (Watanabe et al. 2024).

DM is associated with a decline in cognitive function, which impacts learning, memory, mental processing speed, and cognitive flexibility. Numerous extensive longitudinal investigations have demonstrated that the rate of cognitive loss accelerates in elderly people with DM. However, the specific factors contributing to this accelerated cognitive decline remain unclear (Cheng et al. 2012). Furthermore, patients suffering from DM have 50%–100% increased chances of depression than the overall population, and hospital‐based research and epidemiological surveys have found that the occurrence of DM among bipolar disorder patients is either increased in the overall population or the same (Akhaury and Chaware 2022). Perinatal mental disorders are more prevalent at the time of diagnosis among women suffering from gestational DM but are observed to decrease by late pregnancy and at 6 months after birth. These disorders are more prevalent among women with particular risk factors who may benefit from extra support (Ohene‐Agyei et al. 2024).

Anxiety disorders and severe persistent mental illness, such as schizophrenia, significantly co‐occur in patients with DM, leading to poor mortality and disease outcomes (Schoepf et al. 2012; Ho et al. 2025; Mersha et al. 2022; Albai et al. 2024). Individuals with DM have an increased risk of developing anxiety disorder, which leads to poor glycemic control and reduced treatment adherence while increasing the risk of cardiovascular disease and mortality among DM patients (Mersha et al. 2022; Albai et al. 2024; Chaturvedi et al. 2019; Naicker et al. 2017; Guerrero Fernández de Alba et al. 2020). Conversely, the risk of DM is 19% higher among those with anxiety disorders and increases by two‐ to fivefold in those with SPMIs, including schizophrenia (Schoepf et al. 2012; Ho et al. 2025; Smith et al. 2013; Suvisaari et al. 2016). These frequently co‐occurring mental disorders increase mortality, hospitalization rates, and the risk of adverse complications in patients with DM, increasing the overall disease burden while highlighting the need for targeted mental care interventions in this high‐risk cohort (Ho et al. 2025; Chan et al. 2021). Hence, we conducted a detailed analysis of adults’ death records from the Centers for Disease Control and Prevention‐Wide‐ranging ONline Data for Epidemiologic Research (CDC WONDER) database, listing DM and mental disorders and determining mortality trends across different geographics and demographics from 1999 to 2023 to identify the populations and regions at risk.

## 2 Methods

### 2.1 Study Setting and Population

Data on deaths that took place in the United States related to DM and mental disorders were extracted from the CDC WONDER database (CDC 2017). The Multiple Cause‐of‐Death Public Use record death certificates were studied to identify records in which both DM and mental disorders were mentioned as either contributing or underlying causes of death on nationwide death certificates. This database has previously been used to determine trends in mortality of both DM and mental disorders (Cha and Kim 2021; Beissova et al. 2023; Rivard et al. 2022; Ahmed et al. 2022). DM cases were identified using International Classification of Diseases 10th Revision Clinical Modification (ICD‐10‐CM) codes E10–E14 and mental disorders using codes F01–F99 among decedents aged 35 years and older. Additionally, mental disorders (F01–F99) covered a wide range of mental and behavioral conditions such as depression, anxiety disorders, dementia, schizophrenia, and substance use disorders. This study was exempt from local Institutional Review Board approval because the CDC WONDER database contains publicly available, anonymized data.

### 2.2 Data Extraction

For DM and mental disorders‐related deaths, data for population sizes and location of death (including medical facilities [outpatient, inpatient, and death on arrival and nursing home], decedent home, and other places) were extracted. Demographics (sex, race/ethnicity, and age) and geographical information (urban‐rural, census region, and state) were extracted from 1999 to 2023. Race/ethnicities were defined as non‐Hispanic (NH) White, NH African American, Hispanic or Latino, NH American Indian or Alaskan Native, and NH Asian or Pacific Islander patients. These race/ethnicity categories have previously been used within analyses from the CDC WONDER database and rely on reported data on death certificates. Age groups were defined as 35–44, 45–54, 55–64, 65–74, 75–84, and 85+ years of age. For urban–rural classifications, the 2013 National Center for Health Statistics Urban‐Rural Classification Scheme was used to divide the counties into metropolitan and nonmetropolitan areas (Ingram and Franco 2014). Regions were classified into Northeast, Midwest, South, and West according to the Census Bureau definitions.

### 2.3 Statistical Analysis

Crude mortality rates (CMRs) and age‐adjusted mortality rates (AAMRs) per 100,000 population were measured. CMR was determined by dividing the number of DM + mental disorders‐related deaths by the corresponding US population of that year. AAMRs were calculated by standardizing DM + mental disorders‐related deaths to the year 2000 US Population as previously described (Anderson and Rosenberg 1998). The Joinpoint Regression Program (Joinpoint V 4.9.0.0, National Cancer Institute) was used to determine trends in AAMRs using annual percent change (APC) with 95% confidence interval (CI) (Joinpoint Regression Program n.d.). This software identifies significant variations in AAMRs over time by fitting log‐linear regression models where temporal shifts occurred. APCs were considered increasing or decreasing if the slope describing the change in mortality was significantly different from zero using two‐tailed *t*‐testing. Statistical significance was set at *p* < 0.05.

## 3 Results

### 3.1 Proportional Mortality Rate Across Different Variables From 1999 to 2023

From 1999 to 2023, DM and mental disorders were registered on 1,332,198 death certificates, among which males accounted for 55% of the total demises and the rest, 45%, were females. NH whites constituted a major portion (84%) of overall deaths, followed by NH African Americans (13%), Hispanics or Latinos (7%), and NH Asians or Pacific Islanders (2%), while NH American Indians or Alaska Natives made up only 1% of the entire mortalities. Deaths were the highest among older adults aged 75–84, 85+, and 65–74 years, accounting for 29%, 26%, and 23% of the total deaths and followed by those aged 55–64 (14%), 45–54 (6%), and 35–44 (2%) years, respectively (Table S1).

When considering geographics, metropolitan areas had the highest mortality, representing 77% of the total fatalities, and the rest, 23%, were recorded from nonmetropolitan areas. Peak deaths were reported from the decedent's home (32%), followed by nursing home/long‐term care (28%) > inpatient medical setting (24%) > outpatient or ER facility (7%) > and other places (4%) (Figure S2). Among census regions, the South had the highest mortality, comprising 38% of the total deaths, whereas the regions of the Midwest, West, and Northeast contributed to 27%, 19%, and 17% of the overall deaths (Table S1).

### 3.2 Overall Age‐Adjusted Trends for DM and Mental Disorders‐Related Mortality From 1999 to 2023

**FIGURE 1:**
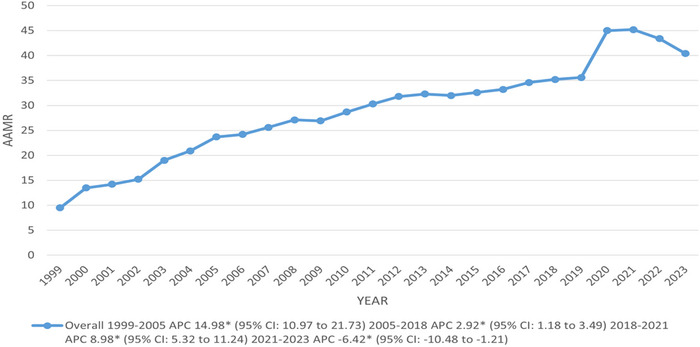
Overall trends in DM and mental disorders‐related age‐adjusted mortality rates per 100,000 among adults aged 35 and above in the United States, 1999–2023. APC = annual percentage change, CI = confidence interval. * The annual percentage change (APC) is significantly different from zero at *α* = 0.05.

A fourfold increase in AAMR was observed, from 9.5 in 1999 to 40.4 in 2023 per 100,000 population. From 1999 to 2005, the AAMR showed a significant increase and continued to rise sharply up to 2018, with associated APCs of 14.98 and 2.92, respectively. However, after reaching its maximum till 2021 (APC: 8.98; 95% CI: 5.32–11.24), the AAMR started to decline significantly up to 2023 (APC: −6.42; 95% CI: −10.48 to −1.21) (Figure 1) (Tables S2 and S3).

### 3.3 Demographic Trends

From 1999 to 2023, notable shifts were seen across different demographic subgroups in the United States.

#### 3.3.1 Gender Stratified

**FIGURE 2:**
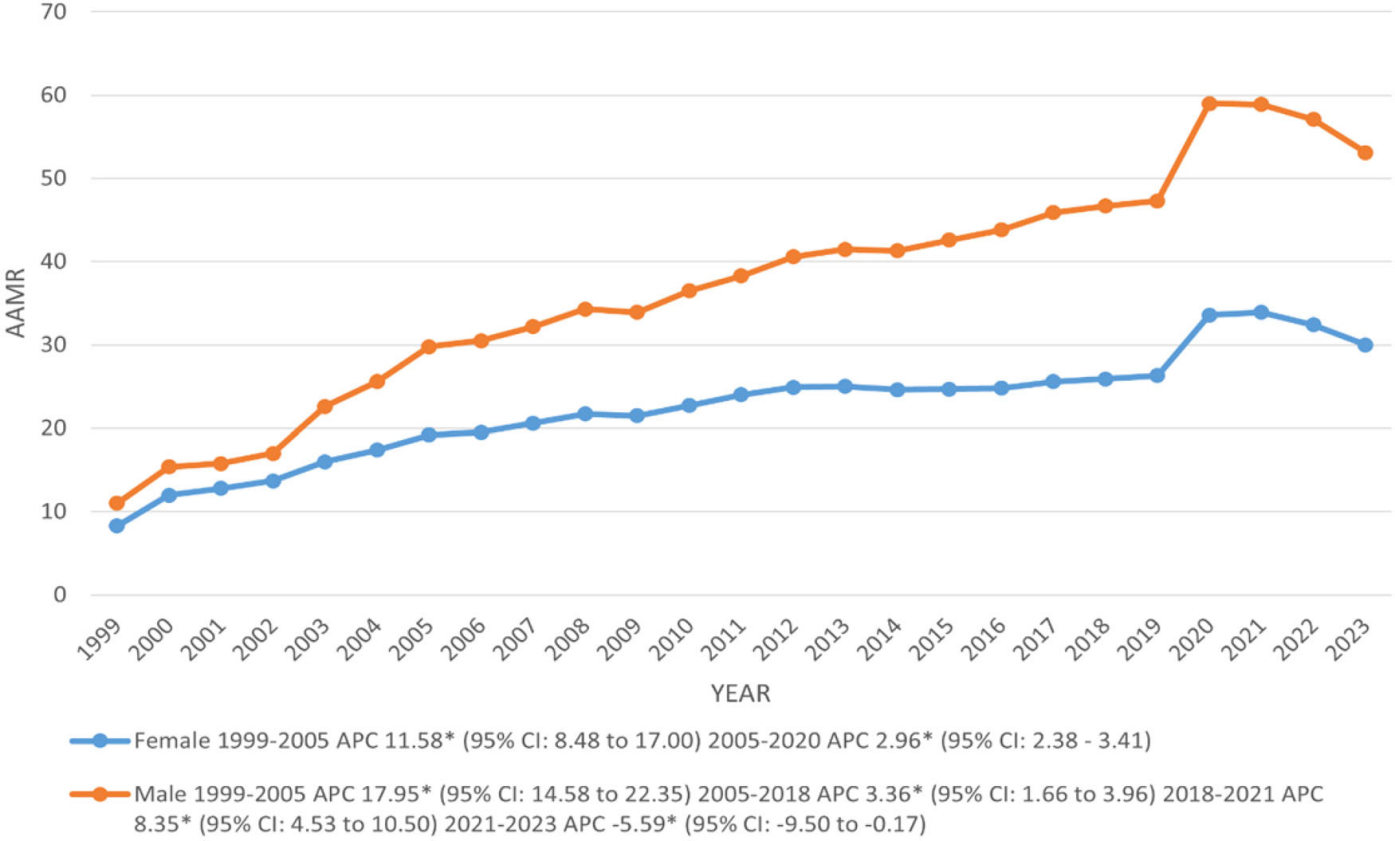
Trends in DM and mental disorders‐related age‐adjusted mortality rates per 100,000, stratified by sex among adults aged 35 and above in the United States, 1999–2023. APC = annual percentage change, CI = confidence interval. * The annual percentage change (APC) is significantly different from zero at *α* = 0.05.

Males had elevated AAMRs per 100,000 population in comparison to females throughout the duration of the study (overall AAMRs: 36.8 males vs. 22.4 females). Similarly, the deaths were higher in males than in females (deaths: males: 735,338 vs. females: 596,860). From 1999, both males and females exhibited a significant rise in AAMR till 2005, with associated APCs of 17.95 and 11.58, respectively. After 2005, the AAMR continued to increase sharply up till 2023 in females (APC: 2.96; 95% CI: 2.38–3.41), while significant surges were seen in males AAMR till 2018, which kept rising sharply till 2021 with associated APCs of 3.36 and 8.35, respectively. However, a prominent decline was recorded in males from 2021 to 2023 (APC: −5.59; 95% CI: −9.50 to −0.17) (Figure 2) (Tables S1–S3).

#### 3.3.2 Race/Ethnicity Stratified

The AAMR (per 100,000 population) was the highest among NH: American Indian or Alaskan Native (39.8) and NH African American (39.6), followed by NH White (28.4) and Hispanic or Latino (24.4), while the lowest was observed in NH Asians or Pacific Islanders (13.7). In contrast, the total deaths varied across different races/ethnicities following that NH White reported the highest number of deaths, totaling 1,117,677, followed by NH African American (173,143), Hispanic or Latino (88,283), and lastly, NH: Asian or Pacific Islander, and American Indian or Alaskan Native reported the least of 25,394 and 14,417 deaths (Tables S1 and S4).

**FIGURE 3:**
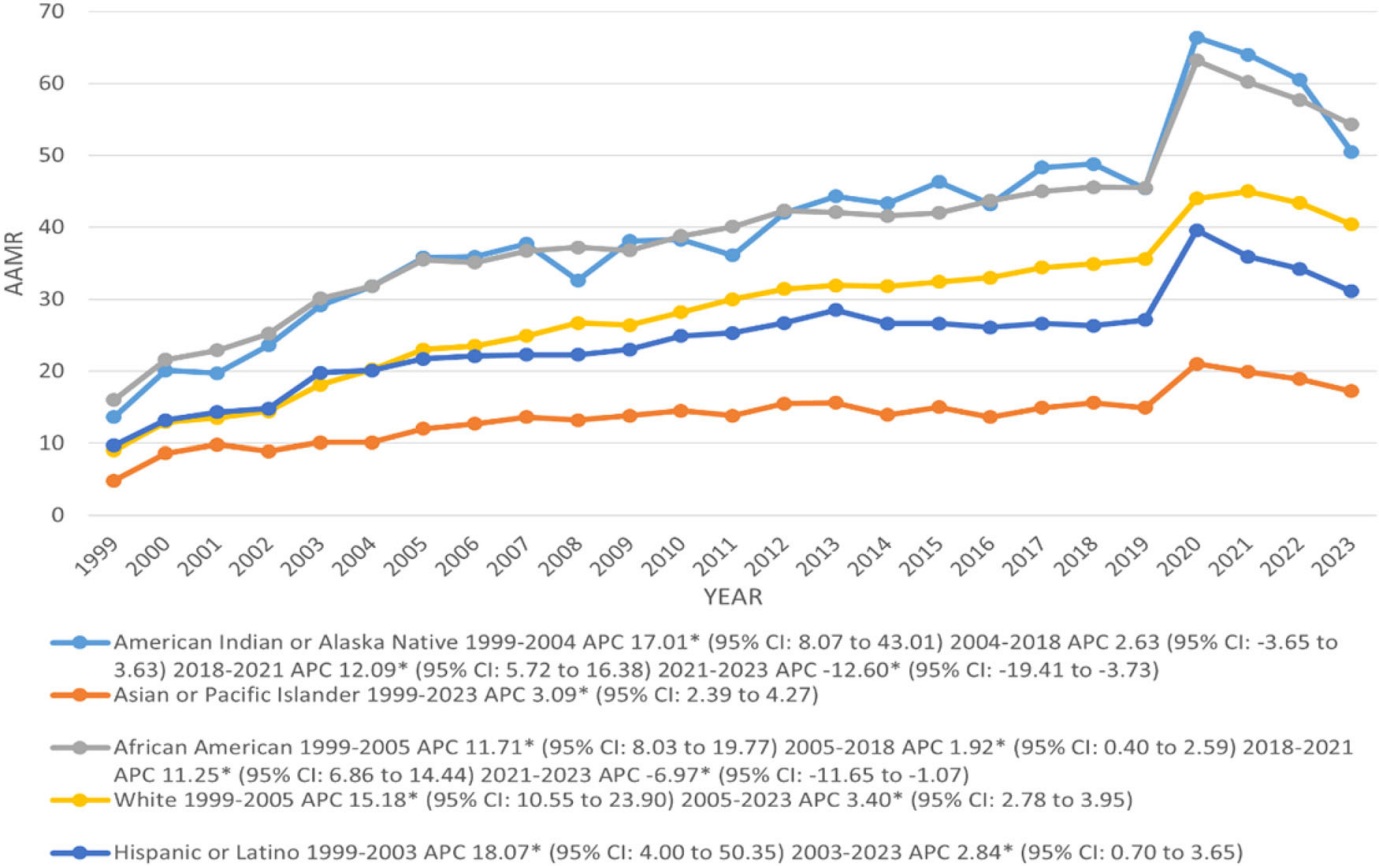
Trends in DM and mental disorders‐related age‐adjusted mortality rates per 100,000, stratified by race and ethnicity among adults aged 35 and above in the United States, 1999–2023. APC = annual percentage change, CI = confidence interval. * The annual percentage change (APC) is significantly different from zero at *α* = 0.05.

A significant incline in AAMR was seen from 1999 to 2003 in Hispanic or Latino (APC: 18.07; 95% CI: 4.00–50.35) and till 2004 in NH American Indian or Alaska Native (APC: 17.01; 95% CI: 8.07–43.01), while it rose up to 2005 in NH: White and African American with associated APCs of 15.18 and 11.71, respectively, whereas in NH Asian or Pacific Islander it increased significantly till 2023 (APC: 3.09; 95% CI: 2.39–4.27). Further, from 2004 and 2005 onward till 2018, the AAMR sharply increased in NH African American and maintained a steady incline in NH American Indian or Alaska Native with associated APCs of 1.92 and 2.63, respectively, and continued to rise significantly till 2021 with corresponding APCs of 11.25 and 12.09; however, a great decline was noticed among both NH: African American and American Indian or Alaska Native from 2021 to 2023 with associated APCs of −6.97 and −12.60, respectively. Furthermore, from 2003 and 2005 onward till 2023, the AAMR increased sharply in Hispanic or Latino and NH White with corresponding APCs of 2.84 and 3.40, respectively (Figure 3) (Table S3).

#### 3.3.3 Age Group Stratified

A consistent upward trend in CMR (per 100,000 population) was observed with the increasing age throughout our study. CMR was 11‐fold higher in older adults (65+) compared with adults aged 35–64 years. Registered deaths were highest in individuals aged 75–84 years, followed by those aged 85+, 65–74, 55–64, 45–54, and 35–44 years, respectively (deaths: 390,125 > 349,463 > 300,981 > 192,359 > 77,837 > 21,433) (Tables S1 and S5).

From 1999, the CMR in individuals aged 35–44 and 85+ years rose sharply up to 2001 and 2003 with associated APCs of 15.16 and 30.92, respectively, and rose significantly up till 2005 in those aged 45–54, 55–64, 65–74, and 75–84 years with corresponding APCs of 17.78, 20.31, 20.62, and 14.82, respectively. By then, the CMR continued to increase significantly till 2023 in those aged 75–84 and 85+ years with corresponding APCs of 2.95 and 3.07, whereas up till 2018 in individuals aged 45–54, 55–64, and 65–74 years with associated APCs of 4.91, 4.60, and 3.18, respectively, a period of stability was noted till 2018 in those aged 35–44 years. Following 2018, the CMR maintained to ascent significantly up to 2021 across these age groups as indicated by APC values: 35–44 (18.67), 45–54 (8.73), 55–64 (9.92), and 65–74 (7.72) years and a great decline was observed till 2023 with corresponding APCs of −9.71, −5.61, ‐6.59 and −6.76, respectively (Figures S1A and S1B) (Table S3).

### 3.4 DM and Mental Disorders‐Related AAMR Stratified by Geographical Variables

**FIGURE 4:**
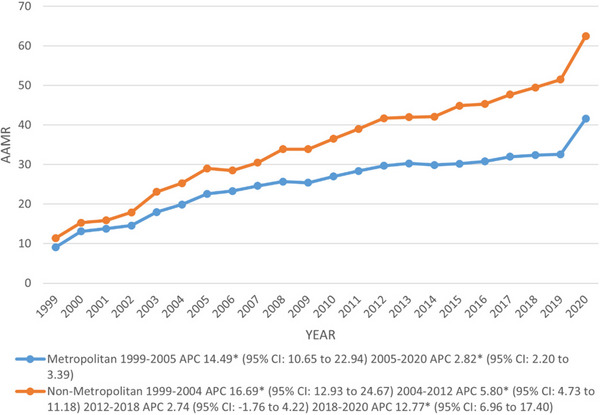
Trends in DM and mental disorders‐related age‐adjusted mortality rates per 100,000, stratified by urbanization among adults aged 35 and above in the United States, 1999–2020. APC = annual percentage change, CI = confidence interval. * The annual percentage change (APC) is significantly different from zero at *α* = 0.05.

Throughout our study, significant variations were detected in AAMRs across diverse geographical subcategories in the US nonmetropolitan areas consistently observed higher AAMR per 100,000 population relative to metropolitan areas (overall AAMR: nonmetropolitan: 35.9 vs. metropolitan: 26.2). Conversely, higher deaths were reported in metropolitan than nonmetropolitan areas (deaths: metropolitan: 817,539 vs. nonmetropolitan: 237,956). The AAMR significantly increased from 1999 to 2004 and 2005 in nonmetropolitan and metropolitan areas with associated APCs of 14.49 and 16.69 and continued to increase sharply up to 2020 in metropolitan areas, whereas up till 2012 in nonmetropolitan areas with associated APCs of 2.82 and 5.8, respectively. Following a period of stability from 2012 to 2018, the AAMR in nonmetropolitan areas was observed to decline significantly till 2020 (APC: 12.77; 95% CI: 6.96–17.40) (Figure 4) (Tables S1, S3, and S6).

**FIGURE 5:**
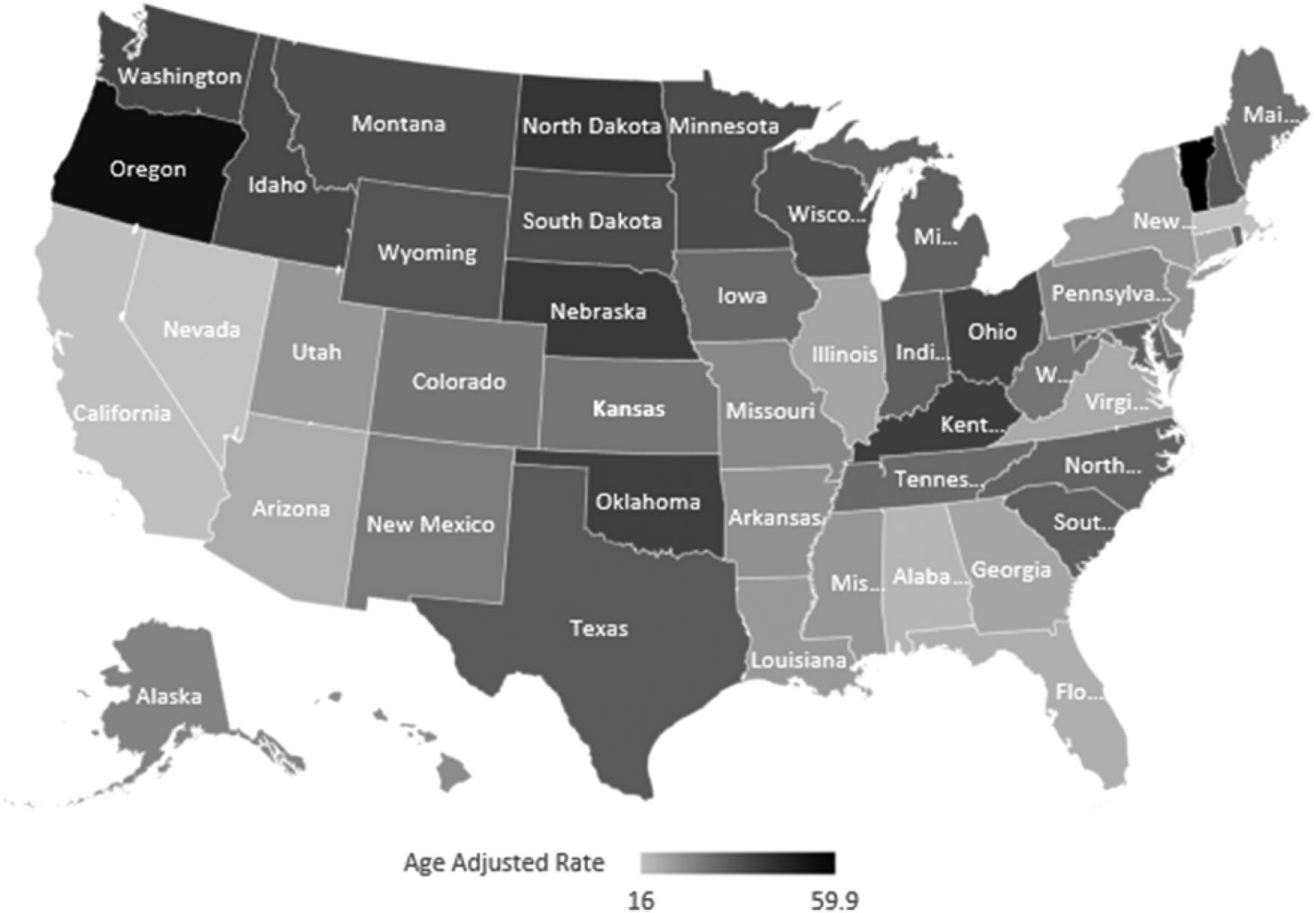
DM and mental disorders‐related age‐adjusted mortality rates per 100,000, stratified by states among adults aged 35 and above in the United States, 1999–2020.

Regionally, the Midwest became apparent with the highest AAMR (34.7) per 100,000 population, followed by the South (29.4). Northeast (25.3), and the region of West displayed the lowest AAMR (24.9). From the states: Vermont stands with the topmost AAMR (59.9), whereas Nevada had the lowest AAMR (Suvisaari et al. 2016). Top 90th percentile states: Vermont, Oregon, North Dakota, Nebraska, and Kentucky had an AAMR nearly three times larger than the AAMR of states Florida, Alabama, California, Massachusetts, and Nevada in the lower 10th percentile (Figure 5) (Tables S7 and S8).

## 4 Discussion

This study aimed to understand the burden of DM and related mental disorders. We computed and refined outputs of 25 years from CDC WONDER databases to examine the AAMRs of mental disorders with an underlying DM present in the population of the United States. Our results revealed a fourfold increase in AAMR with significant rising shifts observed between 1999 and 2021, followed by a period of significant decline till the end of the study. Mortality rates were higher in men, NH American Indians and African Americans ethnicity, nonmetropolitan areas, and the Midwest region for all years through 1999–2023. When looked at more closely, the highest AAMR was observed in Vermont, with deaths being more prevalent in the descendant's home and the nursing care facility. Recognizing mortality trends associated with mental disorders and DM is essential, as these chronic conditions can intensify acute conditions, leading to fatal outcomes. Identifying populations and regions at heightened risk is vital for enhancing healthcare management efficacy in the United States, with the potential for replication in other regions.

Our analysis showed a significant rise in mortality trends between 2018 and 2021, which can be accounted for by the distress brought by COVID‐19, including the crowding of healthcare systems, subsequently the inaccessibility to healthcare and needed medicines, generalized anxiety due to COVID‐19‐related fear, and lockdown‐associated depression (Moradian et al. 2021; Singhai et al. 2020). Individuals with DM and mental disorders had an increased risk of COVID‐related mortality compared to those without these conditions, and the absence of social support during this period, combined with physiological distress, led to increased eating behaviors, with 73% reporting DM mismanagement amid the heightened COVID‐19 crisis (Tang et al. 2023; Vai et al. 2021; Toubasi et al. 2021; Mantovani et al. 2020; Roncon et al. 2020). Further, COVID‐19 was frequently listed as the primary cause of death during a pandemic, overshadowing other contributing conditions such as DM and mental disorders, while our study included only those death records where both DM and mental disorders were mentioned as either primary or contributing causes of death. This helps to capture the overall mortality burden that may have been underreported due to COVID‐related attribution bias, reflecting the contribution of DM and mental disorders associated with mortality during the peak rise between 2018 and 2021. The rates then declined significantly from 2021 till 2023, which could be due to the decreased COVID stress, as by early 2023, hospitalization rates and intensive care unit admissions consistently declined, freeing up the choked healthcare services and allowing greater attention to other diseases like DM (Sarker et al. 2023).

When stratified by gender, it is evident that the male population saw greater rates for our study examination from 1999 to 2023 than the female population. The male‐to‐female mortality ratio has significantly increased, almost doubling, from 1.719 in 1999 to 3.088 in 2020. These numbers are consistent with available literature in the United States, with male mortality greater than female mortality for decades and are linked to increased vulnerability to chronic conditions (Beltrán‐Sánchez et al. 2015). However, this disparity cannot just be attributed to genetic differences but also to exposure, epidemiology, and differences in behaviors of men and women (Yang and Kozloski 2012).

Our results noted a significant difference when examining ethnic or racial groups. Higher AAMR was seen for the American Indian and the African American groups, with marginal differences between both. DM management and self‐care are reported to be lower in African Americans than in the White community (Amoako 2004). Several factors contribute to the aforementioned, including but not limited to cultural beliefs, language/accent barriers, low literacy rates, and medical healthcare disparities (Ricci‐Cabello et al. 2013). American Indians were seen to have the highest prevalence of DM when compared to others, making up almost 30% collectively, although when reviewing the literature, it was found that scientists excluded the group when testing for glucose‐lowering therapies (Rodríguez and Campbell 2017). Not just with DM but with an array of chronic conditions, healthcare disparities were seen to be consistent with research (Chandler and Monnat 2015).

Significant variance in urbanization was evident from our results, with AAMRs higher for nonmetropolitan regions. A ‘neighborhood disadvantage’ was seen affecting chronic condition care like DM in onset and management (Uddin et al. 2022). Our results are consistent with a study in 2023, which reported nonmetropolitan areas to have 22% higher odds of developing DM than metropolitan areas and 14% fewer odds for HbA1c testing, signifying the gaps in preventative care and monitoring of DM in these areas (Hammerslag and Talbert 2023).

Although the results cannot confirm the correlation between DM and mental disorders‐associated mortality, they do affirm and assert it. Similar findings were noted in a study by Soo et al. (2021) in Singapore, which claimed the association of DM with both mild behavioral impairment (MBI) and mild cognitive impairment showed positive for 28.1%, meeting the criteria of having DM and MBI. A meta‐analysis reported that when checking for mental disorder onset in patients with DM than with no DM, almost every DM, including depression, anxiety, schizophrenia, and ADHD, prevalence increased in patients with DM (Benton et al. 2023). Further, a study that pooled 43,992 DM patients showed that patients with diagnosed schizophrenia and bipolar disorder along with DM are more prone to death (Vinogradova et al. 2010). Thus, this strong relationship between DM and mental disorders can be attributed to common biological factors and behavioral mechanisms such as chronic inflammation, insulin resistance, and HPA axis dysfunction linking DM with mental disorders (Ding et al. 2023; Holt et al. 2014; Moulton et al. 2015). Individuals with these conditions have reduced treatment adherence and quality of life, while social factors like poverty, low awareness, and limited access to healthcare further increase the mortality risk, highlighting the need for localized interventions and resource allocation (Guerrero Fernández de Alba et al. 2020; Murillo et al. 2017; Cai et al. 2024).

## 5 Study Limitations

There are several limitations to our study. First, we relied entirely on ICD‐10 code classifications assigned by WHO, which can lead to errors like misinterpretations or omissions. However, ICD‐10 coding is a standard used for global mortality surveillance, which allows consistent tracking and analysis of death data across the world; chart reviews or integrated national health surveys could offer improved diagnostic accuracy, but such data are not available at the considered population level across 25 years. Further covariates, such as patient's age, comorbidities, and socioeconomic background that can highly influence healthcare and mortality outcomes, cannot be analyzed due to the absence of relevant information. Also, the database did not include laboratory and clinical results or treatment histories, making it impossible for us to conduct an in‐depth evaluation. Comorbidities such as cardiovascular disease, chronic kidney disease, and obesity that are common in DM patients can affect the accuracy of cause of death attribution and were not analyzed separately due to the limited nature of the dataset. Lastly, due to the unavailability of data for urbanization and states beyond 2020, recent significant assessments might have been compromised.

## 6 Conclusions

Overall, our investigation illuminates the patterns in the mortality rates of mental disorders associated with DM in the United States from 1999 to 2023. The results show persisting discrepancies, with higher mortality rates among men, NH African Americans, American Indians, and nonmetropolitan residents, who may experience greater impediments to healthcare and chronic disease management. The dramatic increase in mortality during the COVID‐19 pandemic proves the impact of external stressors on chronic disease outcomes, emphasizing the significance of accessible and continuous care. Future studies should investigate methods to enhance access to healthcare for at‐risk groups and delve deeper into the behavioral, socioeconomic, and systemic factors contributing to these trends. We may strive toward more fair healthcare solutions and improved support for those managing mental disorders and DM by addressing these gaps.

## Author Contributions

**Syed Tawassul Hassan**: conceptualization, writing – original draft, writing – review and editing, visualization, data curation, formal analysis, project administration, supervision, software. **Muhammad Shaheer Bin Faheem**: conceptualization, writing – review and editing, writing – original draft, visualization, data curation, formal analysis, supervision, project administration. **Yasmeen Sufi**: writing – original draft, investigation. **Anushah Nadeem**: Resources, validation, visualization. **Wajeeha Siddiqui**: Resources, validation, investigation. **Dur E Sameen**: Resources, methodology, validation. **Amna Kaleem Ahmed**: Resources, methodology, validation. **Faiza Nasir**: Methodology, validation, resources. **Sumaya Samadi**: Resources, project administration.

## Ethics Statement

The authors have nothing to report.

## Consent

The authors have nothing to report.

## Conflicts of Interest

The authors declare no conflicts of interest.

## Peer Review

The peer review history for this article is available at https://publons.com/publon/10.1002/brb3.70723.

## Supporting information




**Supplemental Table 1**. Absolute number of DM and Mental Disorders‐related deaths and percent total deaths among adults aged 35 and above stratified by overall, gender, race/ ethnicity, age group, place of death, urbanization and regions in the U.S., 1999–2023.
**Supplementary Figure 1A**. Trends in DM and Mental Disorders‐related crude mortality rates stratified by age groups 35–64 years (middle‐aged adults) in the U.S., 1999 to 2023. APC = Annual Percentage Change, CI = Confidence Interval. *Indicates that the Annual Percentage Change (APC) is significantly different from zero at α = 0.05.
**Supplementary Figure 1B**. Trends in DM and Mental Disorders‐related crude mortality rates stratified by age groups 65–85+ years (older adults) in the U.S., 1999 to 2023. APC = Annual Percentage Change, CI = Confidence Interval. *Indicates that the Annual Percentage Change (APC) is significantly different from zero at α = 0.05.
**Supplemental Table 2**. Overall and sex‐stratified DM and Mental Disorders‐related age‐adjusted mortality rates per 100,000 among adults aged 35 and above in the U.S., 1999 to 2023.
**Supplementary Figure 2**. Percent total deaths of DM and Mental Disorders‐related by place of deaths among adults aged 35 and above in the U.S., 1999 to 2023.
**Supplemental Table 3**. Annual percent change (APC) of DM and Mental Disorders‐related age‐adjusted mortality rates per 100,000 among adults aged 35 and above in the U.S., 1999 to 2023.
**Supplemental Table 4**. Race/ Ethnicity stratified DM and Mental Disorders‐related age‐adjusted mortality rates per 100,000 among adults aged 35 and above in the U.S., 1999 to 2023.
**Supplemental Table 5**. Age group stratified DM and Mental Disorders‐related crude mortality rates per 100,000 among adults aged 35 and above in the U.S., 1999 to 2023.
**Supplemental Table 6**. Urbanization stratified DM and Mental Disorders‐related age‐adjusted mortality rates per 100,000 among adults aged 35 and above in the U.S., 1999 to 2020.
**Supplemental Table 7**. Region‐stratified DM and Mental Disorders‐related age‐adjusted mortality rates per 100,000 among adults aged 35 and above in the U.S., 1999 to 2023.
**Supplemental Table 8**. State‐stratified DM and Mental Disorders‐related age‐adjusted mortality rates per 100,000 and their respective percentiles among adults aged 35 and above in the U.S., 1999 to 2020.

## Data Availability

The data that support the findings of this study are available in the supplementary material of this article.
