# Supplementary material for: Trends in Diabetes Mellitus and Associated Mental Disorders‐Related Mortality (1999–2023): A CDC WONDER Database Analysis
Source: Brain Behav. 2025 Aug 4;15(8):e70723. doi: 10.1002/brb3.70723 (PMC12321977; doi:10.1002/brb3.70723)
Supplement: Supplementary file 1 — Supplemental Table 1. Absolute number of DM and Mental Disorders‐related deaths and percent total deaths among adults aged 35 and above stratified by overall, gender, race/ ethnicity, age group, place of death, urbanization and regions in the U.S., 1999–2023. Supplementary Figure 1A. Trends in DM and Mental Disorders‐related crude mortality rates stratified by age groups 35–64 years (middle‐aged adults) in the U.S., 1999 to 2023. APC = Annual Percentage Change, CI = Confidence Interval. *Indicates that the Annual Percentage Change (APC) is significantly different from zero at α = 0.05. Supplementary Figure 1B. Trends in DM and Mental Disorders‐related crude mortality rates stratified by age groups 65–85+ years (older adults) in the U.S., 1999 to 2023. APC = Annual Percentage Change, CI = Confidence Interval. *Indicates that the Annual Percentage Change (APC) is significantly different from zero at α = 0.05. Supplemental Table 2. Overall and sex‐stratified DM and Mental Disorders‐related age‐adjusted mortality rates per 100,000 among adults aged 35 and above in the U.S., 1999 to 2023. Supplementary Figure 2. Percent total deaths of DM and Mental Disorders‐related by place of deaths among adults aged 35 and above in the U.S., 1999 to 2023. Supplemental Table 3. Annual percent change (APC) of DM and Mental Disorders‐related age‐adjusted mortality rates per 100,000 among adults aged 35 and above in the U.S., 1999 to 2023. Supplemental Table 4. Race/ Ethnicity stratified DM and Mental Disorders‐related age‐adjusted mortality rates per 100,000 among adults aged 35 and above in the U.S., 1999 to 2023. Supplemental Table 5. Age group stratified DM and Mental Disorders‐related crude mortality rates per 100,000 among adults aged 35 and above in the U.S., 1999 to 2023. Supplemental Table 6. Urbanization stratified DM and Mental Disorders‐related age‐adjusted mortality rates per 100,000 among adults aged 35 and above in the U.S., 1999 to 2020. Supplemental Table 7. Region‐strat [file BRB3-15-e70723-s001.docx]

**SUPPLEMENTARY FIGURE/TABLE LEGENDS**

**Supplemental Table 1.** Absolute number of DM and Mental Disorders-related deaths and percent total deaths among adults aged 35 and above stratified by overall, gender, race/ ethnicity, age group, place of death, urbanization and regions in the U.S., 1999-2023.

**Supplementary Figure 1A.** Trends in DM and Mental Disorders-related crude mortality rates stratified by age groups 35-64 years (middle-aged adults) in the U.S., 1999 to 2023.

APC = Annual Percentage Change, CI = Confidence Interval.

*Indicates that the Annual Percentage Change (APC) is significantly different from zero at α = 0.05.

**Supplementary Figure 1B.** Trends in DM and Mental Disorders-related crude mortality rates stratified by age groups 65-85+ years (older adults) in the U.S., 1999 to 2023.

APC = Annual Percentage Change, CI = Confidence Interval.

*Indicates that the Annual Percentage Change (APC) is significantly different from zero at α = 0.05.

**Supplemental Table 2.** Overall and sex-stratified DM and Mental Disorders-related age-adjusted mortality rates per 100,000 among adults aged 35 and above in the U.S., 1999 to 2023.

**Supplementary Figure 2.** Percent total deaths of DM and Mental Disorders-related by place of deaths among adults aged 35 and above in the U.S., 1999 to 2023.

**Supplemental Table 3.** Annual percent change (APC) of DM and Mental Disorders-related age-adjusted mortality rates per 100,000 among adults aged 35 and above in the U.S., 1999 to 2023.

**Supplemental Table 4.** Race/ Ethnicity stratified DM and Mental Disorders-related age-adjusted mortality rates per 100,000 among adults aged 35 and above in the U.S., 1999 to 2023.

**Supplemental Table 5.** Age group stratified DM and Mental Disorders-related crude mortality rates per 100,000 among adults aged 35 and above in the U.S., 1999 to 2023.

**Supplemental Table 6.** Urbanization stratified DM and Mental Disorders-related age-adjusted mortality rates per 100,000 among adults aged 35 and above in the U.S., 1999 to 2020.

**Supplemental Table 7.** Region-stratified DM and Mental Disorders-related age-adjusted mortality rates per 100,000 among adults aged 35 and above in the U.S., 1999 to 2023.

**Supplemental Table 8.** State-stratified DM and Mental Disorders-related age-adjusted mortality rates per 100,000 and their respective percentiles among adults aged 35 and above in the U.S., 1999 to 2020.

**SUPPLEMENTARY FIGURES/TABLES:**

| **Variables** | **Deaths** | **% of Total Deaths** |
| --- | --- | --- |
| Overall | 1332198 | 100% |
| **Gender** | | |
| Female | 596860 | 45% |
| Male | 735338 | 55% |
| **Race** | | |
| NH White | 1117677 | 84% |
| NH African American | 173143 | 13% |
| NH Asian or Pacific Islander | 25394 | 2% |
| NH American Indian or Alaskan Native | 14417 | 1% |
| Hispanic or Latino | 88283 | 7% |
| **Age Groups** | | |
| 35-44 years | 21433 | 2% |
| 45-54 years | 77837 | 6% |
| 55-64 years | 192359 | 14% |
| 65-74 years | 300981 | 23% |
| 75-84 years | 390125 | 29% |
| 85+ years | 349463 | 26% |
| **Place of Deaths** | | |
| Medical Facility - Inpatient | 314316 | 24% |
| Medical Facility - Outpatient or ER | 92202 | 7% |
| Medical Facility - Dead on Arrival | 6595 | 0% |
| Decedent's home | 430392 | 32% |
| Nursing home/long term care | 373537 | 28% |
| Other | 57205 | 4% |
| **Urbanization** | | |
| Metropolitan Areas | 817539 | 77% |
| Non-Metropolitan Areas | 237956 | 23% |
| **Regions** | | |
| Northeast | 225287 | 17% |
| Midwest | 356024 | 27% |
| South | 502347 | 38% |
| West | 248540 | 19% |
| **NH: Non-Hispanic** | | |

**Supplemental Table 1.** Absolute number of DM and Mental Health Disorders-related deaths and percent total deaths among adults aged 35 and above stratified by overall, gender, race/ ethnicity, age group, place of death, urbanization and regions in the United States, 1999-2023.

**
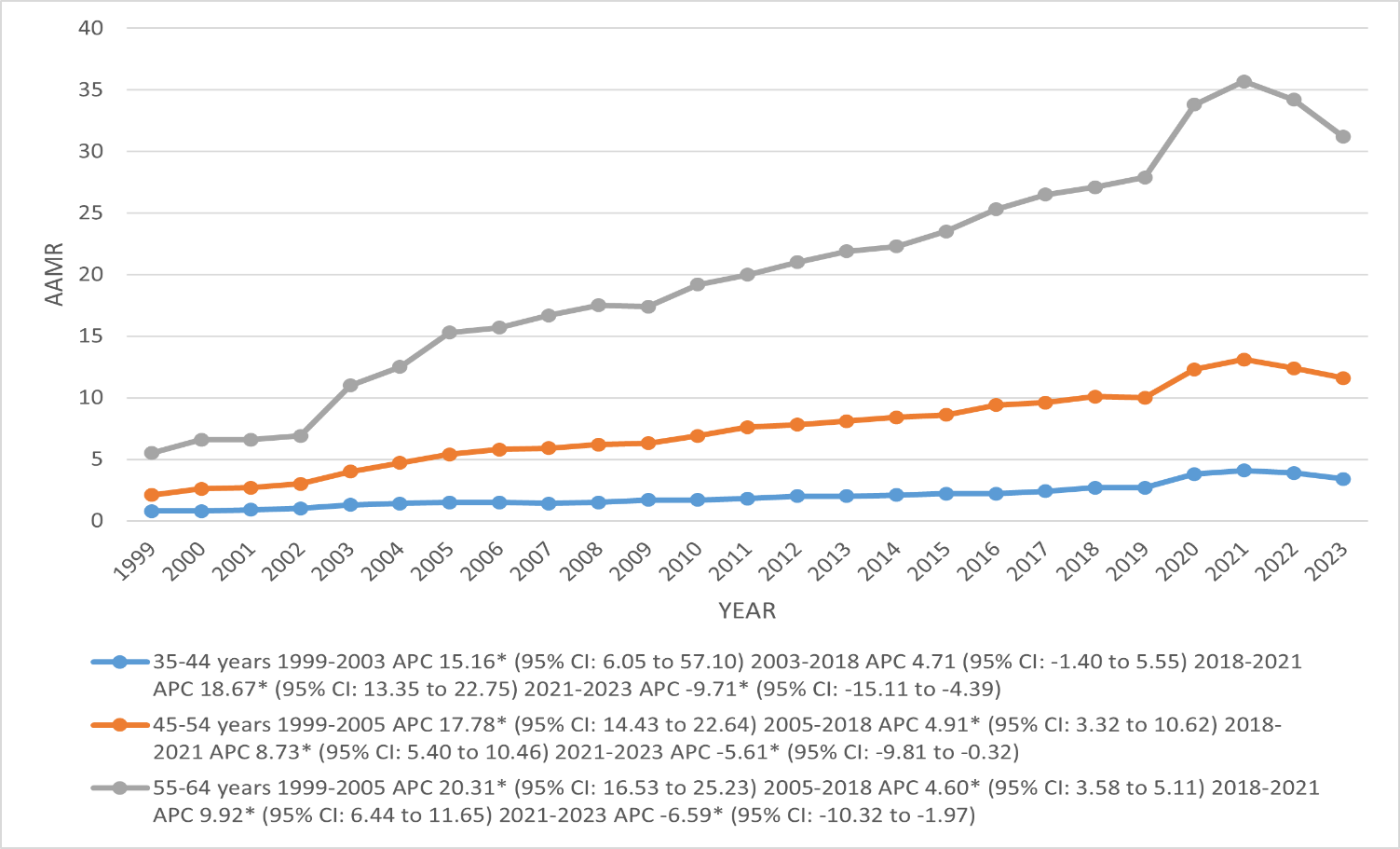
**

**Supplementary Figure 1A.** Trends in DM and Mental Health Disorders-related crude mortality rates stratified by age groups 35-64 years (middle-aged adults) in the United States, 1999 to 2023.

APC = Annual Percentage Change, CI = Confidence Interval.

*Indicates that the Annual Percentage Change (APC) is significantly different from zero at α = 0.05.


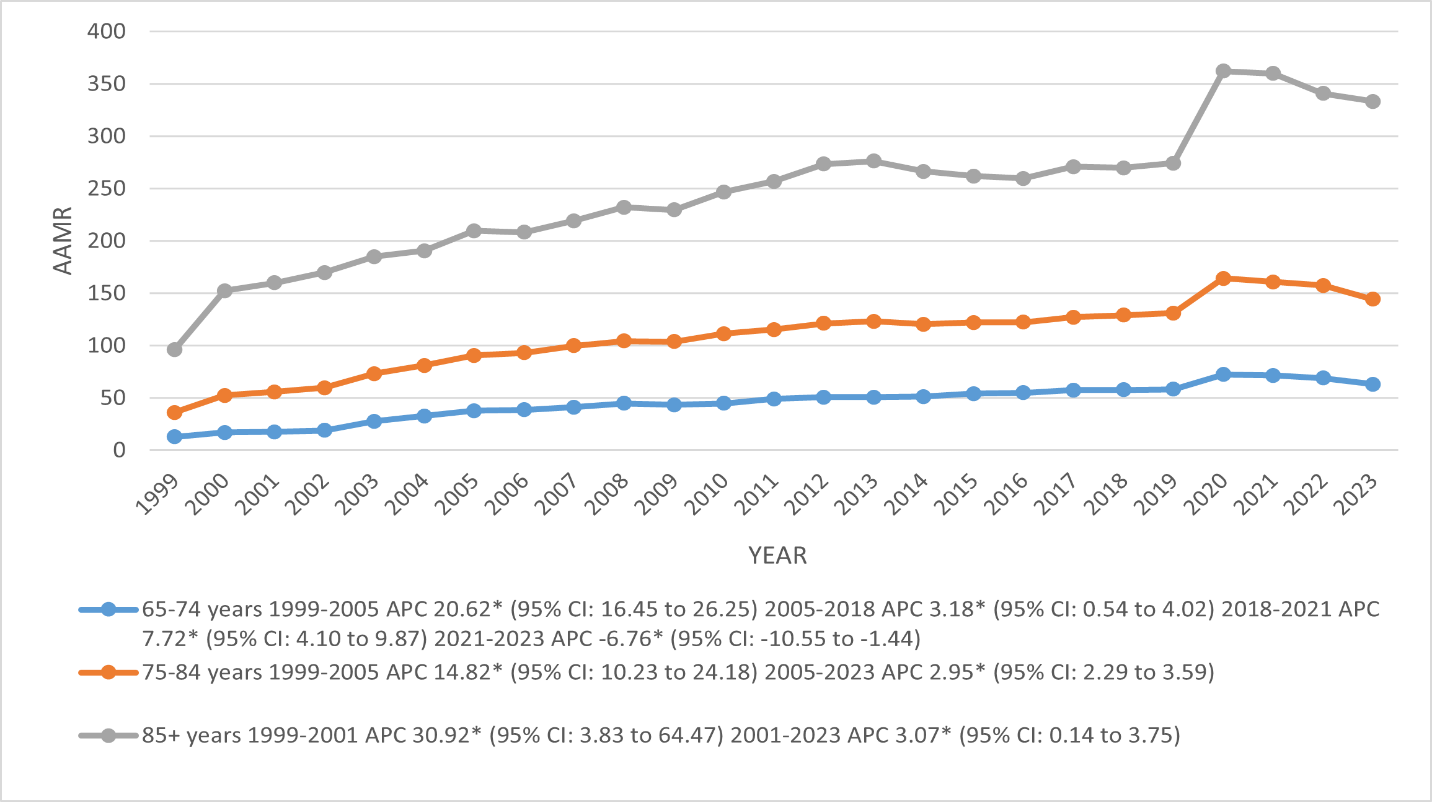


**Supplementary Figure 1B.** Trends in DM and Mental Health Disorders-related crude mortality rates stratified by age groups 65-85+ years (older adults) in the United States, 1999 to 2023.

APC = Annual Percentage Change, CI = Confidence Interval.

*Indicates that the Annual Percentage Change (APC) is significantly different from zero at α = 0.05.

| **Age-Adjusted Rate /100,000 (95% CI)** | | | |
| --- | --- | --- | --- |
| **Year** | **Overall** | **Male** | **Female** |
| 1999 | 9.5 (9.4 - 9.7) | 11 (10.7 - 11.3) | 8.3 (8.1 - 8.5) |
| 2000 | 13.5 (13.3 - 13.7) | 15.4 (15.1 - 15.7) | 12 (11.8 - 12.3) |
| 2001 | 14.2 (14 - 14.4) | 15.8 (15.5 - 16.2) | 12.8 (12.6 - 13) |
| 2002 | 15.2 (15 - 15.4) | 17 (16.6 - 17.3) | 13.7 (13.5 - 14) |
| 2003 | 19 (18.8 - 19.2) | 22.6 (22.2 - 23) | 16 (15.8 - 16.3) |
| 2004 | 20.9 (20.7 - 21.2) | 25.6 (25.2 - 26) | 17.4 (17.1 - 17.6) |
| 2005 | 23.7 (23.5 - 24) | 29.8 (29.3 - 30.2) | 19.2 (18.9 - 19.5) |
| 2006 | 24.2 (23.9 - 24.4) | 30.5 (30 - 30.9) | 19.5 (19.2 - 19.8) |
| 2007 | 25.6 (25.4 - 25.9) | 32.2 (31.7 - 32.6) | 20.6 (20.3 - 20.9) |
| 2008 | 27.1 (26.9 - 27.4) | 34.3 (33.9 - 34.8) | 21.7 (21.4 - 22) |
| 2009 | 26.9 (26.7 - 27.2) | 33.9 (33.4 - 34.3) | 21.5 (21.2 - 21.8) |
| 2010 | 28.7 (28.5 - 29) | 36.5 (36 - 36.9) | 22.7 (22.4 - 23) |
| 2011 | 30.3 (30 - 30.5) | 38.3 (37.9 - 38.8) | 24 (23.7 - 24.3) |
| 2012 | 31.8 (31.5 - 32.1) | 40.6 (40.2 - 41.1) | 24.9 (24.6 - 25.2) |
| 2013 | 32.3 (32 - 32.6) | 41.5 (41 - 41.9) | 25 (24.7 - 25.3) |
| 2014 | 32 (31.7 - 32.3) | 41.3 (40.8 - 41.7) | 24.6 (24.3 - 24.9) |
| 2015 | 32.6 (32.4 - 32.9) | 42.6 (42.2 - 43.1) | 24.7 (24.4 - 25) |
| 2016 | 33.2 (33 - 33.5) | 43.8 (43.3 - 44.2) | 24.8 (24.5 - 25.1) |
| 2017 | 34.6 (34.4 - 34.9) | 45.9 (45.5 - 46.4) | 25.6 (25.3 - 25.9) |
| 2018 | 35.2 (34.9 - 35.4) | 46.7 (46.3 - 47.2) | 25.9 (25.6 - 26.2) |
| 2019 | 35.6 (35.4 - 35.9) | 47.3 (46.9 - 47.8) | 26.3 (26 - 26.6) |
| 2020 | 45 (44.7 - 45.3) | 59 (58.5 - 59.5) | 33.6 (33.2 - 33.9) |
| 2021 | 45.2 (44.9 - 45.5) | 58.9 (58.4 - 59.4) | 33.9 (33.6 - 34.2) |
| 2022 | 43.4 (43.2 - 43.7) | 57.1 (56.6 - 57.6) | 32.4 (32.1 - 32.8) |
| 2023 | 40.4 (40.2 - 40.7) | 53.1 (52.6 - 53.5) | 30 (29.7 - 30.3) |
| **Total** | 28.8 (28.6 - 29.1) | 36.8 (36.4 - 37.3) | 22.4 (22.2 - 22.7) |

**Supplemental Table 2.** Overall and sex-stratified DM and Mental Health Disorders-related age-adjusted mortality rates per 100,000 among adults aged 35 and above in the United States, 1999 to 2023.


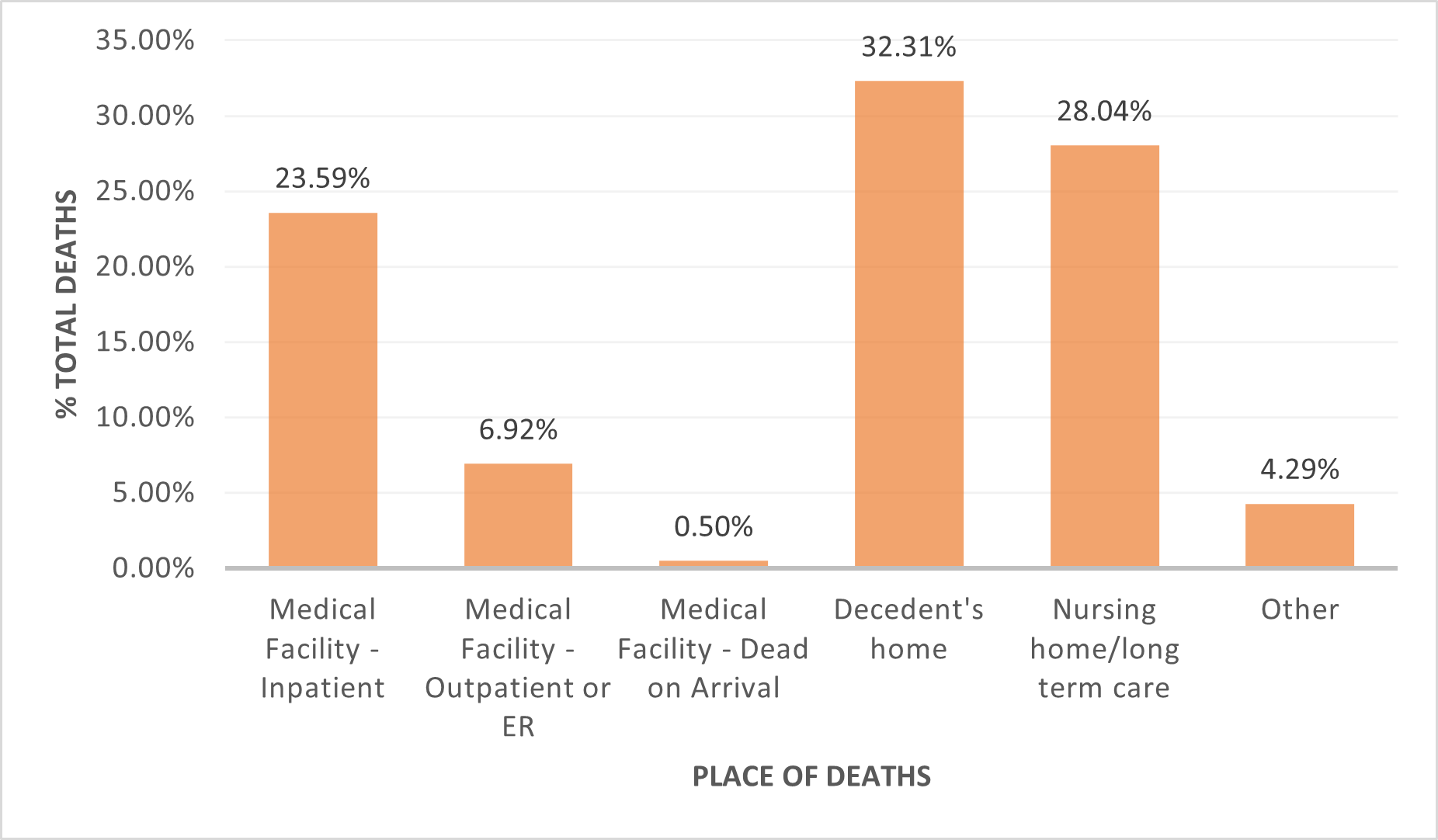


**Supplementary Figure 2.** Percent total deaths of DM and Mental Health Disorders-related by place of deaths among adults aged 35 and above in the United States, 1999 to 2023.

| **Year Interval** | **APC (95% CI)** |
| --- | --- |
| **Overall** | |
| 1999-2005 | 14.98* (10.97 - 21.73) |
| 2005-2018 | 2.92* (1.18 - 3.49) |
| 2018-2021 | 8.98* (5.32 - 11.24) |
| 2021-2023 | -6.42* (-10.48 - -1.21) |
| **Gender** | |
| **Male** | |
| 1999-2005 | 17.95* (14.58 - 22.35) |
| 2005-2018 | 3.36* (1.66 - 3.96) |
| 2018-2021 | 8.35* (4.53 - 10.50) |
| 2021-2023 | -5.59* (-9.50 - -0.17) |
| **Female** | |
| 1999-2005 | 11.58* (8.48 - 17.00) |
| 2005-2023 | 2.96* (2.38 - 3.41) |
| **Race** | |
| **NH American Indian or Alaska Native** | |
| 1999-2004 | 17.01* (8.07 - 43.01) |
| 2004-2018 | 2.63 (-3.65 - 3.63) |
| 2018-2021 | 12.09* (5.72 - 16.38) |
| 2021-2023 | -12.60* (-19.41 - -3.73) |
| **NH Asian or Pacific Islander** | |
| 1999-2023 | 3.09* (2.39 - 4.27) |
| **NH African American** | |
| 1999-2005 | 11.71* (8.03 - 19.77) |
| 2005-2018 | 1.92* (0.40 - 2.59) |
| 2018-2021 | 11.25* (6.86 - 14.44) |
| 2021-2023 | -6.97* (-11.65 - -1.07) |
| **NH White** | |
| 1999-2005 | 15.18* (10.55 - 23.90) |
| 2005-2023 | 3.40* (2.78 - 3.95) |
| **Hispanic or Latino** | |
| 1999-2003 | 18.07* (4.00 - 50.35) |
| 2003-2023 | 2.84* (0.70 - 3.65) |
| **Age Groups** | |
| **35-44 years** | |
| 1999-2003 | 15.16* (6.05 - 57.10) |
| 2003-2018 | 4.71 (-1.40 - 5.55) |
| 2018-2021 | 18.67* (13.35 - 22.75) |
| 2021-2023 | -9.71* (-15.11 - -4.39) |
| **45-54 years** | |
| 1999-2005 | 17.78* (14.43 - 22.64) |
| 2005-2018 | 4.91* (3.32 - 10.62) |
| 2018-2021 | 8.73* (5.40 - 10.46) |
| 2021-2023 | -5.61* (-9.81 - -0.32) |
| **55-64 years** | |
| 1999-2005 | 20.31* (16.53 - 25.23) |
| 2005-2018 | 4.60* (3.58 - 5.11) |
| 2018-2021 | 9.92* (6.44 - 11.65) |
| 2021-2023 | -6.59* (-10.32 - -1.97) |
| **65-74 years** | |
| 1999-2005 | 20.62* (16.45 - 26.25) |
| 2005-2018 | 3.18* (0.54 - 4.02) |
| 2018-2021 | 7.72* (4.10 - 9.87) |
| 2021-2023 | -6.76* (-10.55 - -1.44) |
| **75-84 years** | |
| 1999-2005 | 14.82* (10.23 - 24.18) |
| 2005-2023 | 2.95* (2.29 - 3.59) |
| **85+ years** | |
| 1999-2001 | 30.92* (3.83 - 64.47) |
| 2001-2023 | 3.07* (0.14 - 3.75) |
| **Urbanization** | |
| **Metropolitan** | |
| 1999-2005 | 14.49* (10.65 - 22.94) |
| 2005-2020 | 2.82* (2.20 - 3.39) |
| **Non-Metropolitan** | |
| 1999-2004 | 16.69* (12.93 - 24.67) |
| 2004-2012 | 5.80* (4.73 - 11.18) |
| 2012-2018 | 2.74 (-1.76 - 4.22) |
| 2018-2020 | 12.77* (6.96 - 17.40) |
| **Regions** | |
| **Northeast** | |
| 1999-2006 | 15.83* (11.78 - 24.38) |
| 2006-2023 | 2.12* (1.43 - 2.80) |
| **Midwest** | |
| 1999-2008 | 12.43* (9.87 - 16.53) |
| 2008-2023 | 2.51* (1.73 - 3.24) |
| **South** | |
| 1999-2004 | 15.68* (11.86 - 21.99) |
| 2004-2018 | 3.62* (2.80 - 4.16) |
| 2018-2021 | 11.58* (8.03 - 14.00) |
| 2021-2023 | -5.99* (-9.45 - -1.18) |
| **West** | |
| 1999-2004 | 15.50* (11.47 - 21.17) |
| 2004-2018 | 2.16* (1.13 - 2.82) |
| 2018-2021 | 9.81* (5.74 - 12.34) |
| 2021-2023 | -6.10* (-10.49 - -0.80) |
| **NH: Non-Hispanic; APC: Annual Percent Change** | |

**Supplemental Table 3.** Annual percent change (APC) of DM and Mental Health Disorders-related age-adjusted mortality rates per 100,000 among adults aged 35 and above in the United States, 1999 to 2023.

| **Age-Adjusted Rate /100,000 (95% CI)** | | | | | |
| --- | --- | --- | --- | --- | --- |
| **Year** | **NH American Indian or Alaska Native** | **NH Asian or Pacific Islander** | **NH African American** | **NH White** | **Hispanics or Latino** |
| 1999 | 13.6 (10.8 - 16.9) | 4.8 (3.9 - 5.6) | 16 (15.3 - 16.7) | 9 (8.8 - 9.2) | 9.7 (9 - 10.5) |
| 2000 | 20.1 (16.6 - 23.6) | 8.6 (7.5 - 9.7) | 21.6 (20.7 - 22.4) | 12.9 (12.7 - 13.1) | 13.2 (12.3 - 14.1) |
| 2001 | 19.7 (16.2 - 23.1) | 9.8 (8.7 - 10.9) | 22.9 (22 - 23.8) | 13.5 (13.2 - 13.7) | 14.3 (13.4 - 15.2) |
| 2002 | 23.6 (20 - 27.3) | 8.9 (7.9 - 9.9) | 25.2 (24.3 - 26.1) | 14.4 (14.1 - 14.6) | 14.8 (13.9 - 15.7) |
| 2003 | 29.1 (25.2 - 33) | 10.1 (9 - 11.1) | 30.1 (29.1 - 31.1) | 18.1 (17.9 - 18.3) | 19.8 (18.8 - 20.8) |
| 2004 | 31.8 (27.9 - 35.8) | 10.1 (9 - 11.1) | 31.8 (30.8 - 32.8) | 20.2 (19.9 - 20.4) | 20.1 (19.1 - 21.1) |
| 2005 | 35.8 (31.7 - 39.9) | 12 (10.9 - 13.1) | 35.5 (34.4 - 36.5) | 23 (22.7 - 23.2) | 21.7 (20.7 - 22.7) |
| 2006 | 35.9 (31.9 - 39.9) | 12.7 (11.7 - 13.8) | 35.1 (34 - 36.1) | 23.5 (23.2 - 23.7) | 22.1 (21.1 - 23.1) |
| 2007 | 37.7 (33.6 - 41.8) | 13.6 (12.5 - 14.7) | 36.7 (35.7 - 37.7) | 24.9 (24.7 - 25.2) | 22.3 (21.3 - 23.2) |
| 2008 | 32.6 (29 - 36.2) | 13.2 (12.2 - 14.2) | 37.2 (36.2 - 38.2) | 26.7 (26.4 - 27) | 22.3 (21.4 - 23.3) |
| 2009 | 38.1 (34.2 - 41.9) | 13.8 (12.8 - 14.8) | 36.8 (35.8 - 37.8) | 26.4 (26.1 - 26.6) | 23 (22.1 - 23.9) |
| 2010 | 38.3 (34.4 - 42.1) | 14.5 (13.5 - 15.5) | 38.8 (37.8 - 39.9) | 28.2 (28 - 28.5) | 24.9 (24 - 25.9) |
| 2011 | 36.1 (32.6 - 39.6) | 13.8 (12.9 - 14.8) | 40.1 (39.1 - 41.1) | 30 (29.7 - 30.2) | 25.3 (24.4 - 26.2) |
| 2012 | 42 (38.3 - 45.7) | 15.5 (14.5 - 16.5) | 42.3 (41.2 - 43.3) | 31.4 (31.1 - 31.7) | 26.7 (25.8 - 27.6) |
| 2013 | 44.3 (40.6 - 48.1) | 15.6 (14.7 - 16.5) | 42.1 (41.1 - 43.1) | 31.9 (31.7 - 32.2) | 28.5 (27.6 - 29.4) |
| 2014 | 43.3 (39.7 - 46.9) | 13.9 (13 - 14.7) | 41.6 (40.7 - 42.6) | 31.8 (31.5 - 32) | 26.6 (25.7 - 27.4) |
| 2015 | 46.3 (42.8 - 49.9) | 15 (14.1 - 15.8) | 42 (41 - 42.9) | 32.4 (32.1 - 32.7) | 26.6 (25.8 - 27.5) |
| 2016 | 43.2 (39.9 - 46.6) | 13.6 (12.8 - 14.4) | 43.7 (42.7 - 44.6) | 33 (32.7 - 33.3) | 26.1 (25.3 - 26.9) |
| 2017 | 48.3 (44.8 - 51.8) | 14.9 (14.1 - 15.7) | 45 (44 - 46) | 34.4 (34.1 - 34.7) | 26.6 (25.8 - 27.3) |
| 2018 | 48.8 (45.4 - 52.2) | 15.6 (14.9 - 16.4) | 45.6 (44.7 - 46.6) | 34.9 (34.6 - 35.2) | 26.3 (25.5 - 27.1) |
| 2019 | 45.4 (42.2 - 48.6) | 14.9 (14.1 - 15.6) | 45.5 (44.6 - 46.4) | 35.6 (35.3 - 35.9) | 27.1 (26.3 - 27.8) |
| 2020 | 66.4 (62.6 - 70.1) | 21 (20.2 - 21.9) | 63.2 (62.1 - 64.3) | 44 (43.7 - 44.3) | 39.6 (38.7 - 40.5) |
| 2021 | 64 (60.2 - 67.8) | 19.9 (19 - 20.8) | 60.2 (59.1 - 61.3) | 45 (44.7 - 45.3) | 35.9 (35.1 - 36.8) |
| 2022 | 60.5 (57 - 64.1) | 18.9 (18.1 - 19.7) | 57.7 (56.7 - 58.8) | 43.4 (43.1 - 43.7) | 34.2 (33.4 - 35) |
| 2023 | 50.5 (47.3 - 53.7) | 17.2 (16.4 - 17.9) | 54.3 (53.3 - 55.3) | 40.4 (40.1 - 40.7) | 31.1 (30.3 - 31.9) |
| **Total** | 39.8 (36.2 - 43.5) | 13.7 (12.7 - 14.6) | 39.6 (38.7 - 40.6) | 28.4 (28.1 - 28.6) | 24.4 (23.5- 25.2) |
| **NH: Non-Hispanic** | | | | | |

**Supplemental Table 4.** Race/ Ethnicity stratified DM and Mental Health Disorders-related age-adjusted mortality rates per 100,000 among adults aged 35 and above in the United States, 1999 to 2023.

| **Crude Rate /100,000 (95% CI)** | | | | | | |
| --- | --- | --- | --- | --- | --- | --- |
| **Year** | **35-44 years** | **45-54 years** | **55-64 years** | **65-74 years** | **75-84 years** | **85+ years** |
| 1999 | 0.8 (0.7 - 0.9) | 2.1 (2 - 2.3) | 5.5 (5.2 - 5.8) | 12.9 (12.4 - 13.4) | 36.3 (35.2 - 37.4) | 96.5 (93.5 - 99.5) |
| 2000 | 0.8 (0.7 - 0.9) | 2.6 (2.5 - 2.8) | 6.6 (6.3 - 6.9) | 17.1 (16.5 - 17.7) | 52.6 (51.3 - 53.8) | 152.5 (148.8 - 156.3) |
| 2001 | 0.9 (0.8 - 1) | 2.7 (2.5 - 2.8) | 6.6 (6.3 - 7) | 17.7 (17.1 - 18.3) | 56.1 (54.7 - 57.4) | 160.1 (156.3 - 163.8) |
| 2002 | 1 (0.9 - 1.1) | 3 (2.9 - 3.2) | 6.9 (6.6 - 7.3) | 19.1 (18.5 - 19.7) | 60 (58.6 - 61.3) | 169.9 (166.1 - 173.8) |
| 2003 | 1.3 (1.1 - 1.4) | 4 (3.8 - 4.2) | 11 (10.6 - 11.4) | 27.7 (26.9 - 28.5) | 73.3 (71.8 - 74.8) | 185.1 (181.1 - 189) |
| 2004 | 1.4 (1.3 - 1.5) | 4.7 (4.5 - 4.9) | 12.5 (12.1 - 12.9) | 32.7 (31.9 - 33.5) | 81 (79.5 - 82.6) | 190.9 (186.9 - 194.9) |
| 2005 | 1.5 (1.4 - 1.6) | 5.4 (5.2 - 5.6) | 15.3 (14.8 - 15.7) | 38.1 (37.3 - 39) | 90.6 (88.9 - 92.2) | 209.7 (205.6 - 213.8) |
| 2006 | 1.5 (1.4 - 1.6) | 5.8 (5.6 - 6) | 15.7 (15.2 - 16.1) | 38.7 (37.9 - 39.6) | 93.2 (91.6 - 94.9) | 208.4 (204.4 - 212.5) |
| 2007 | 1.4 (1.3 - 1.6) | 5.9 (5.7 - 6.1) | 16.7 (16.2 - 17.1) | 41.3 (40.4 - 42.2) | 100 (98.3 - 101.7) | 219.5 (215.4 - 223.6) |
| 2008 | 1.5 (1.4 - 1.6) | 6.2 (6 - 6.5) | 17.5 (17.1 - 18) | 45 (44.1 - 45.9) | 104.5 (102.7 - 106.2) | 232.4 (228.2 - 236.5) |
| 2009 | 1.7 (1.5 - 1.8) | 6.3 (6.1 - 6.6) | 17.4 (17 - 17.8) | 43.6 (42.7 - 44.5) | 104 (102.3 - 105.8) | 229.7 (225.7 - 233.8) |
| 2010 | 1.7 (1.5 - 1.8) | 6.9 (6.6 - 7.1) | 19.2 (18.7 - 19.6) | 45 (44.1 - 45.9) | 111.4 (109.6 - 113.2) | 246.7 (242.6 - 250.9) |
| 2011 | 1.8 (1.7 - 1.9) | 7.6 (7.3 - 7.8) | 20 (19.6 - 20.5) | 49 (48.1 - 49.9) | 115.6 (113.8 - 117.5) | 257 (252.8 - 261.1) |
| 2012 | 2 (1.8 - 2.1) | 7.8 (7.6 - 8.1) | 21 (20.5 - 21.4) | 50.8 (49.9 - 51.7) | 121.4 (119.5 - 123.3) | 273.5 (269.3 - 277.7) |
| 2013 | 2 (1.9 - 2.1) | 8.1 (7.8 - 8.4) | 21.9 (21.4 - 22.3) | 50.8 (50 - 51.7) | 123.5 (121.6 - 125.3) | 276.3 (272.1 - 280.4) |
| 2014 | 2.1 (1.9 - 2.2) | 8.4 (8.1 - 8.6) | 22.3 (21.9 - 22.8) | 51.4 (50.5 - 52.3) | 120.5 (118.7 - 122.4) | 266.5 (262.4 - 270.6) |
| 2015 | 2.2 (2.1 - 2.4) | 8.6 (8.3 - 8.9) | 23.5 (23 - 24) | 54.2 (53.4 - 55.1) | 122 (120.2 - 123.9) | 261.9 (257.9 - 265.9) |
| 2016 | 2.2 (2 - 2.3) | 9.4 (9.1 - 9.6) | 25.3 (24.8 - 25.8) | 55 (54.1 - 55.8) | 122.4 (120.6 - 124.2) | 259.7 (255.8 - 263.7) |
| 2017 | 2.4 (2.2 - 2.5) | 9.6 (9.3 - 9.9) | 26.5 (26 - 27) | 57.5 (56.6 - 58.3) | 127.2 (125.4 - 129.1) | 271.1 (267.1 - 275.1) |
| 2018 | 2.7 (2.5 - 2.9) | 10.1 (9.8 - 10.5) | 27.1 (26.6 - 27.6) | 57.9 (57 - 58.7) | 129.2 (127.4 - 131) | 270 (266 - 273.9) |
| 2019 | 2.7 (2.6 - 2.9) | 10 (9.7 - 10.3) | 27.9 (27.4 - 28.4) | 58.5 (57.7 - 59.4) | 131 (129.2 - 132.7) | 274.3 (270.3 - 278.3) |
| 2020 | 3.8 (3.6 - 3.9) | 12.3 (12 - 12.6) | 33.8 (33.2 - 34.3) | 72.4 (71.5 - 73.3) | 164.3 (162.3 - 166.2) | 362.2 (357.6 - 366.8) |
| 2021 | 4.1 (3.9 - 4.3) | 13.1 (12.8 - 13.5) | 35.7 (35.1 - 36.3) | 71.7 (70.8 - 72.6) | 161.1 (159.2 - 163.1) | 360 (355.2 - 364.8) |
| 2022 | 3.9 (3.7 - 4.1) | 12.4 (12 - 12.7) | 34.2 (33.7 - 34.8) | 69.1 (68.2 - 70) | 157.4 (155.6 - 159.3) | 341 (336.5 - 345.5) |
| 2023 | 3.4 (3.2 - 3.6) | 11.6 (11.2 - 11.9) | 31.2 (30.7 - 31.7) | 63.4 (62.5 - 64.2) | 144.3 (142.6 - 146) | 333.5 (328.9 - 338) |
| **Total** | 2.0 (1.9 - 2.2) | 7.4 (7.1 - 7.6) | 20.1 (19.6 - 20.5) | 45.6 (44.8 - 46.4) | 108.1 (106.4 - 109.8) | 244.3 (240.3 - 248.4) |

**Supplemental Table 5.** Age group stratified DM and Mental Health Disorders-related crude mortality rates per 100,000 among adults aged 35 and above in the United States, 1999 to 2023.

| **Age-Adjusted Rate /100,000 (95% CI)** | | |
| --- | --- | --- |
| **Year** | **Metropolitan** | **Non-Metropolitan** |
| 1999 | 9.1 (8.9 - 9.2) | 11.4 (11 - 11.8) |
| 2000 | 13.1 (12.9 - 13.3) | 15.3 (14.8 - 15.7) |
| 2001 | 13.8 (13.6 - 14) | 15.9 (15.5 - 16.4) |
| 2002 | 14.6 (14.3 - 14.8) | 17.9 (17.4 - 18.4) |
| 2003 | 18 (17.8 - 18.3) | 23.1 (22.5 - 23.6) |
| 2004 | 19.9 (19.7 - 20.2) | 25.3 (24.8 - 25.9) |
| 2005 | 22.6 (22.3 - 22.8) | 29 (28.4 - 29.7) |
| 2006 | 23.3 (23 - 23.5) | 28.5 (27.9 - 29.1) |
| 2007 | 24.6 (24.3 - 24.8) | 30.5 (29.9 - 31.2) |
| 2008 | 25.7 (25.4 - 26) | 33.9 (33.2 - 34.6) |
| 2009 | 25.4 (25.1 - 25.6) | 33.9 (33.2 - 34.6) |
| 2010 | 27 (26.8 - 27.3) | 36.5 (35.8 - 37.1) |
| 2011 | 28.4 (28.2 - 28.7) | 39 (38.3 - 39.7) |
| 2012 | 29.7 (29.5 - 30) | 41.7 (40.9 - 42.4) |
| 2013 | 30.3 (30 - 30.6) | 42 (41.2 - 42.7) |
| 2014 | 29.9 (29.6 - 30.2) | 42.1 (41.4 - 42.9) |
| 2015 | 30.2 (29.9 - 30.4) | 44.9 (44.2 - 45.7) |
| 2016 | 30.8 (30.5 - 31.1) | 45.3 (44.5 - 46) |
| 2017 | 32 (31.8 - 32.3) | 47.7 (47 - 48.5) |
| 2018 | 32.4 (32.1 - 32.6) | 49.5 (48.8 - 50.3) |
| 2019 | 32.6 (32.3 - 32.8) | 51.5 (50.7 - 52.3) |
| 2020 | 41.6 (41.3 - 41.9) | 62.5 (61.7 - 63.4) |
| **Total** | 26.2 (26.2 - 26.3) | 35.9 (35.7 - 36) |

**Supplemental Table 6.** Urbanization stratified DM and Mental Health Disorders-related age-adjusted mortality rates per 100,000 among adults aged 35 and above in the United States, 1999 to 2020.

| **Age-Adjusted Rate /100,000 (95% CI)** | | | | |
| --- | --- | --- | --- | --- |
| **Year** | **Northeast** | **Midwest** | **South** | **West** |
| 1999 | 7.9 (7.6 - 8.2) | 10.5 (10.1 - 10.8) | 10.5 (10.2 - 10.8) | 8.4 (8 - 8.7) |
| 2000 | 11.5 (11.1 - 11.9) | 15 (14.6 - 15.4) | 13.6 (13.3 - 14) | 13.5 (13.1 - 14) |
| 2001 | 11.9 (11.5 - 12.3) | 15.8 (15.3 - 16.2) | 14.2 (13.9 - 14.5) | 14.7 (14.2 - 15.1) |
| 2002 | 12.4 (12 - 12.8) | 17 (16.6 - 17.5) | 15.4 (15.1 - 15.8) | 15.3 (14.9 - 15.8) |
| 2003 | 15.9 (15.4 - 16.3) | 19.6 (19.1 - 20.1) | 20.5 (20.1 - 20.8) | 18.5 (18.1 - 19) |
| 2004 | 19.5 (19 - 20) | 23 (22.5 - 23.5) | 20.3 (19.9 - 20.6) | 21.1 (20.6 - 21.7) |
| 2005 | 21.3 (20.8 - 21.8) | 25.7 (25.2 - 26.3) | 24.6 (24.1 - 25) | 22.5 (21.9 - 23) |
| 2006 | 25.1 (24.5 - 25.6) | 25.7 (25.1 - 26.2) | 23.8 (23.3 - 24.2) | 22.5 (21.9 - 23) |
| 2007 | 25.9 (25.3 - 26.4) | 30 (29.4 - 30.6) | 24.4 (24 - 24.8) | 22.9 (22.4 - 23.4) |
| 2008 | 25.9 (25.3 - 26.4) | 35.1 (34.5 - 35.7) | 25.2 (24.7 - 25.6) | 23.3 (22.7 - 23.8) |
| 2009 | 26.1 (25.5 - 26.6) | 34.7 (34.1 - 35.3) | 25.1 (24.7 - 25.5) | 22.5 (22 - 23) |
| 2010 | 26.7 (26.1 - 27.3) | 36.1 (35.5 - 36.7) | 27.6 (27.2 - 28) | 24.7 (24.2 - 25.2) |
| 2011 | 27.8 (27.2 - 28.4) | 39.7 (39 - 40.3) | 28.6 (28.2 - 29) | 25.6 (25.1 - 26.1) |
| 2012 | 28.3 (27.7 - 28.9) | 40.8 (40.2 - 41.4) | 31 (30.6 - 31.4) | 27 (26.4 - 27.5) |
| 2013 | 28.7 (28.2 - 29.3) | 41 (40.4 - 41.7) | 31.7 (31.3 - 32.2) | 27.5 (27 - 28) |
| 2014 | 28.8 (28.2 - 29.3) | 41.4 (40.8 - 42) | 31.5 (31 - 31.9) | 26.1 (25.6 - 26.6) |
| 2015 | 29.1 (28.5 - 29.6) | 41.3 (40.6 - 41.9) | 32.6 (32.2 - 33) | 27.2 (26.7 - 27.7) |
| 2016 | 29 (28.4 - 29.5) | 41.5 (40.9 - 42.1) | 33.6 (33.1 - 34) | 28 (27.5 - 28.5) |
| 2017 | 29.2 (28.6 - 29.7) | 43.4 (42.8 - 44) | 35.3 (34.9 - 35.7) | 29.4 (28.9 - 30) |
| 2018 | 29.9 (29.4 - 30.5) | 42.8 (42.2 - 43.4) | 37.2 (36.7 - 37.6) | 28.7 (28.2 - 29.2) |
| 2019 | 29.9 (29.4 - 30.5) | 42.8 (42.2 - 43.4) | 38 (37.6 - 38.4) | 29.5 (29 - 30) |
| 2020 | 39.1 (38.4 - 39.7) | 54.7 (54 - 55.4) | 47.8 (47.3 - 48.3) | 35.7 (35.1 - 36.2) |
| 2021 | 35.5 (34.9 - 36.1) | 53.3 (52.7 - 54) | 49.4 (48.9 - 49.9) | 38.3 (37.8 - 38.9) |
| 2022 | 34.8 (34.2 - 35.4) | 50.4 (49.7 - 51) | 47.4 (47 - 47.9) | 37.3 (36.7 - 37.8) |
| 2023 | 32.1 (31.6 - 32.7) | 46.9 (46.3 - 47.5) | 45.1 (44.6 - 45.5) | 33.4 (32.9 - 34) |
| **Total** | 25.3 (24.8 - 25.8) | 34.7 (34.2 - 35.3) | 29.4 (29.0 - 29.8) | 24.9 (24.4 - 25.4) |

**Supplemental Table 7.** Region-stratified DM and Mental Health Disorders-related age-adjusted mortality rates per 100,000 among adults aged 35 and above in the United States, 1999 to 2023.

| **State** | **Age-Adjusted Rate /100,000 (95% CI)** | **Percentile** |
| --- | --- | --- |
| Nevada | 16 | 0% |
| Massachusetts | 16.2 | 2% |
| California | 16.4 | 4% |
| Alabama | 18.1 | 6% |
| Florida | 19.4 | 8% |
| Arizona | 19.9 | 10% |
| Virginia | 20.4 | 12% |
| Connecticut | 21 | 14% |
| Georgia | 21.2 | 16% |
| Illinois | 21.3 | 18% |
| New York | 23.2 | 20% |
| Louisiana | 23.6 | 22% |
| Mississippi | 23.9 | 24% |
| Utah | 23.9 | 24% |
| New Jersey | 24.6 | 28% |
| Arkansas | 25.1 | 30% |
| Hawaii | 25.9 | 32% |
| Missouri | 26.5 | 34% |
| Pennsylvania | 27.2 | 36% |
| Alaska | 27.7 | 38% |
| District of Columbia | 29.1 | 40% |
| New Mexico | 29.2 | 42% |
| Colorado | 29.9 | 44% |
| Delaware | 30 | 46% |
| Kansas | 30.4 | 48% |
| West Virginia | 30.4 | 48% |
| Maine | 31.8 | 52% |
| Tennessee | 32.5 | 54% |
| Iowa | 32.9 | 56% |
| North Carolina | 34.1 | 58% |
| Rhode Island | 34.2 | 60% |
| Maryland | 34.5 | 62% |
| Indiana | 34.7 | 64% |
| Michigan | 34.7 | 64% |
| South Carolina | 35 | 68% |
| Texas | 36.2 | 70% |
| New Hampshire | 36.9 | 72% |
| Wyoming | 37.4 | 74% |
| Wisconsin | 38.3 | 76% |
| Montana | 38.9 | 78% |
| Minnesota | 39.4 | 80% |
| South Dakota | 39.4 | 80% |
| Idaho | 39.8 | 84% |
| Washington | 40.4 | 86% |
| Ohio | 41.7 | 88% |
| Oklahoma | 42 | 90% |
| Kentucky | 42.5 | 92% |
| Nebraska | 43.8 | 94% |
| North Dakota | 44.7 | 96% |
| Oregon | 55.6 | 98% |
| Vermont | 59.9 | 100% |

**Supplemental Table 8.** State-stratified DM and Mental Health Disorders-related age-adjusted mortality rates per 100,000 and their respective percentiles among adults aged 35 and above in the United States, 1999 to 2020.
